# Age-stratified association of weight-adjusted waist index with grip strength in community-dwelling older adults in China: The mediating effect of appendicular skeletal muscle mass

**DOI:** 10.1097/MD.0000000000049929

**Published:** 2026-07-24

**Authors:** Yujin Wang, Ying Gao, Yi Li, Yingying Wu

**Affiliations:** a School of Nursing, Inner Mongolia Medical University, Hohhot, Inner Mongolia, China; b Department of Nursing, Hohhot First Hospital, Hohhot, Inner Mongolia, China.

**Keywords:** grip strength, mediating effect, sarcopenia, skeletal muscle mass of the limbs, weight-adjusted-waist index

## Abstract

The weight-adjusted waist index (WWI) serves as an emerging metric for evaluating body fat distribution, but its association with muscle strength in older adults and the potential mediating role of appendicular skeletal muscle mass (ASM) remain unclear, particularly across different age groups. This research sought to elucidate the association between WWI and grip strength in the elderly community population, with a particular focus on determining whether ASM mediates this relationship and how these dynamics differ by age. This cross-sectional, community-based study recruited 204 adults aged ≥60 years from communities in Hohhot, China. The study population was divided into 2 age-based strata: 60 to 70 years, n = 137 and >70 years, n = 67. The WWI was derived by normalizing waist circumference against the square root of total body mass. ASM was estimated utilizing bioelectrical impedance analysis, while grip strength of the dominant hand was quantified via an electronic dynamometer. To evaluate whether ASM acted as a mediator, mediation analysis was carried out using the PROCESS macro with 5000 bootstrap iterations. In the 60- to 70-age group, a positive correlation linking WWI to grip strength was found, which was completely driven by ASM (indirect effect = 1.739, 95% confidence interval: 1.028–2.611). In the >70 age group, ASM partially accounted for the inverse relationship observed between WWI and grip strength (indirect effect = −0.507, 95% confidence interval: −1.313 to −0.006). ASM acts as a crucial intermediary in the age-dependent link between central adiposity and muscle strength among older adults living in the community.WWI may be a practical, age-specific indicator for sarcopenia risk screening in community health settings.

## 1. Introduction

As the global population ages, age-related skeletal muscle disorders have emerged as a significant public health concern. As a systemic and advancing skeletal muscle disorder, sarcopenia entails a multifaceted deterioration encompassing diminished muscle mass, weakened strength, and impaired physical function. Grip strength is extensively acknowledged as a reliable proxy for overall muscular function and an effective prognostic marker for detrimental health events. Current literature consistently highlights that muscle depletion and weakness among seniors act as critical risk factors for adverse outcomes such as falls, fractures, loss of independence, and mortality, thereby imposing a dual burden on both individual life satisfaction and public health systems.^[1]^ Traditional adiposity indices, such as body mass index (BMI), have important limitations in older populations because they do not distinguish between fat and lean masses. Consequently, conditions such as sarcopenic obesity and normal-weight obesity may be misclassified.^[2]^ Therefore, the identification of novel indicators that more accurately reflect body fat distribution and its relationship with muscle health is of considerable importance.

Among the emerging anthropometric measures, the weight-adjusted waist index (WWI) has attracted increasing attention as a novel metric for evaluating obesity. This was first proposed by Park et al.^[3]^ WWI is derived by taking the ratio of waist circumference (WC) to the square root of total body mass. This metric provides a refined estimate of visceral fat accumulation by minimizing the interference of overall weight in evaluating central obesity. Furthermore, existing literature indicates that WWI is correlated with cardiometabolic hazards and mortality from any cause, potentially exhibiting superior performance compared with traditional indicators such as BMI.^[4]^ In populations with chronic conditions, including diabetes and osteoporosis, Sarcopenia has been linked to elevated levels of the WWI, underscoring its relevance to muscle–fat interactions.^[5,6]^ However, the associations between WWI and muscle strength and ASM in community-dwelling older adults remain insufficiently characterized. In particular, it is unclear whether these relationships vary with age.

There exists significant heterogeneity in how WWI relates to muscle strength, with this relationship shifting as individuals advance in age. In relatively younger older adults, greater body mass may contribute to increased mechanical loading, thereby enhancing muscle function. In contrast, in more advanced age, excess fat accumulation and age-related anabolic resistance may adversely affect muscle strength. Existing studies have reported inconsistent findings, demonstrating both positive and negative associations, and have seldom accounted for age as a potential effect modifier.^[1,7]^

Therefore, this study aimed to investigate the associations among WWI, appendicular skeletal muscle mass (ASM), and grip strength in community-dwelling older adults, with particular emphasis on age-related heterogeneity. Specifically, this study contrasted these indices across the 60 to 70 and >70 age brackets, analyzed the link between WWI and grip strength, and determined whether ASM acts as a mediator. These findings may help clarify the age-specific relevance of WWI as an indicator of muscle health and provide evidence to support stratified strategies for identifying the risk of sarcopenia in older populations.

## 2. Methods

### 2.1. Design

Data were collected from 204 older adults (≥60 years) living in Hohhot communities during the period of September 2024 through September 2025. Ethical approval for this study was granted by the institutional review board of the participating hospital (No. IRB2024302). The research was performed in strict adherence to the principles outlined in the Declaration of Helsinki (2013), and all participants provided written informed consent.

### 2.2. Sample and setting

The sample size of 204 participants was determined based on the feasibility of recruitment within the designated study period (September 2024 to 2025). All eligible older adults who met the inclusion criteria were consecutively enrolled. Eligible older adults were identified and recruited with the assistance of community health service centers, using public notices and face-to-face invitations. To assess whether this sample size was adequate, we applied a commonly accepted rule of thumb for cross-sectional studies, which recommends at least 10 participants per independent variable. The present study included up to 12 independent variables in the planned analyses, requiring a minimum of 120 participants. With 204 participants, the sample exceeded this minimum threshold, suggesting sufficient statistical power.

Participants were enrolled based on the following criteria: age of 60 years or older, residency in Hohhot for a minimum of 6 months, a mini-mental state examination score of 24 or higher, and voluntary agreement confirmed by written informed consent.

Conversely, individuals were excluded if they presented with severe cardiac, hepatic, or renal impairment, specifically defined as New York Heart Association class III–IV heart failure, Child–Pugh class B–C cirrhosis, or an estimated glomerular filtration rate below 30 mL/min/1.73 m^2^; acute systemic infection or advanced malignancy (stage IV according to the TNM classification); contraindications to bioelectrical impedance analysis (BIA), including the presence of a cardiac pacemaker or metallic implants; limb disability or unhealed fracture precluding accurate assessment of grip strength; and participation in structured resistance training (≥2 sessions per week) or use of anabolic hormonal agents within the preceding 3 months.

The study population was categorized into 2 distinct age-based cohorts(60–70 and >70 years) according to established gerontological frameworks, which indicate differences in physiological reserve and comorbidity profiles across these age categories.

### 2.3. Measures

#### 2.3.1. General information

Demographic and lifestyle characteristics were obtained using a structured questionnaire, including age, sex, place of residence, educational attainment, income level, marital status, living status, smoking behavior, and alcohol use.

#### 2.3.2. Anthropometric measurements and calculation of the WWI

Following standardized protocols, we obtained anthropometric measurements including height, weight, and WC from subjects dressed in light clothing without shoes.

BMI was derived by dividing weight (kg) by the square of height (m^2^). Furthermore, WWI was computed as WC (cm) divided by the square root of weight (kg).^[3]^ The association between WWI and the study outcomes was evaluated using continuous variables as well as quartile stratification.

#### 2.3.3. Skeletal muscle mass in the limbs

BIA was utilized to assess ASM, a noninvasive and widely applied method with good reproducibility for body composition assessment.^[8]^ Measurements were performed using an InBody analyzer under standardized conditions, including fasting status, an empty bladder, and the removal of metallic items. ASM was calculated as the combined muscle mass of the upper and lower extremities (kg).

#### 2.3.4. Grip strength

Grip strength measurement is one of the gold standards for diagnosing sarcopenia, and its reliability and predictive validity have been established in numerous international studies.^[9]^ In this study, grip strength was assessed using a handheld dynamometer, an effective method for evaluating upper-limb muscle strength and overall muscle health. The measurement protocol was as follows: for each hand, 2 trials were conducted, and the highest reading was retained. We defined low grip strength based on the Asian Working Group for Sarcopenia thresholds, which are <28 kg for men and <18 kg for women.^[10]^

### 2.4. Quality control

To guarantee the consistency and reliability of the data, we implemented several quality assurance protocols. All investigators received unified training in interview techniques, study procedures, and questionnaire administration. Trained interviewers were responsible for gathering data using face-to-face methods. Data entry was performed independently by 2 researchers, followed by verification and reconciliation of discrepancies to minimize input errors.

### 2.5. Statistical methods

We utilized SPSS software (version 27.0; IBM Corp., Armonk, New York, USA) for all statistical computations. The Shapiro–Wilk test was applied to evaluate the distribution of the data. Continuous data are summarized as means ± SD or medians (interquartile ranges) based on their distribution, with group differences assessed via independent-samples *t*-tests or Mann–Whitney *U* tests. Categorical variables are reported as frequencies (percentages) and analyzed using the *χ*^2^ test or Fisher exact test, where applicable. Stratified regression analyses were conducted to explore the link connecting WWI with grip strength. The PROCESS macro (model 4) was used to test mediation effects, relying on 5000 bootstrap resamples to derive 95% confidence intervals (CIs). The mediation effect was considered significant when the bias-corrected CI excluded 0. A *P*-value of <.05 (2-tailed) indicated statistical significance.

## 3. Result

### 3.1. Demographic data

This study included 204 older individuals living in the community, comprising 137 participants aged 60 to 70 years and 67 participants aged >70 years. Age differed significantly between the groups (*t* = −22.453, *P* < .001), educational level (*χ*^2^ = 9.936, *P* = .007), WWI (*t* = 2.345, *P* = .020), ASM (*Z* = −2.489, *P* *=* .013), and grip strength (*Z* = −2.312, *P* = .021). Participants aged >70 years had lower WWI scores, less ASM, and weaker grip strength. Table 1 indicates that sex, residence, marital status, and history of smoking or alcohol use were comparable between the groups (all *P* > .05).

**Table 1:** Baseline characteristics of the surveyed population.

Variable	Total sample (n = 204)	60 ≤ Age ≤ 70 (n = 137)	Age > 70 (n = 67)	*t/χ* ^*2*^ */Z*	*P*
Age [(years), *X̄* ± s]	68.08 ± 6.00	64.54 ± 3.00	75.31 ± 3.62	‐22.453	<.001
Gender				0.878	.349
Male [n (%)]	76 (37.3)	48 (35.0)	28 (41.8)		
Female [n (%)]	128 (62.7)	89 (65.0)	39 (58.2)		
Current residence				0.232	.630
Urban [n (%)]	196 (96.1)	131 (95.6)	65 (97.0)		
Rural [n (%)]	8 (3.9)	6 (4.4)	2 (3.0)		
Educational attainment [n (%)]				9.936	.007
Elementary school or below	49 (24.0)	30 (21.9)	19 (28.4)		
Junior high school	72 (35.3)	41 (29.9)	31 (46.3)		
High school and above	83 (40.7)	66 (48.2)	17 (25.4)		
Average monthly household income [n (%)]				1.298	.730
<3000 Yuan	86 (42.2)	61 (44.5)	25 (37.3)		
3000–6999 Yuan	99 (48.5)	64 (46.7)	35 (52.2)		
7000–9999 Yuan	15 (7.4)	9 (6.6)	6 (9.0)		
≥10,000 Yuan	4 (2.0)	3 (2.2)	1 (1.5)		
Marital status [n (%)]				2.081	.149
Married	180 (88.2)	124 (90.5)	56 (83.6)		
Unmarried (unmarried/divorced/widowed)	24	13 (9.5)	11 (16.4)		
Living arrangements [n (%)]				3.430	.330
Living alone	18 (8.8)	11 (8.0)	7 (10.4)		
With spouse	163 (79.9)	107 (78.1)	56 (83.6)		
Children	20 (9.8)	16 (11.7)	4 (6.0)		
Other	3 (1.5)	3 (2.2)	0 (0.0)		
Smoking status [n (%)]				3.858	.145
Nonsmokers	172 (84.3)	112 (81.8)	60 (89.6)		
Smokers	25 (12.3)	21 (15.3)	4 (6.0)		
Former smokers	7 (3.4)	4 (2.9)	3 (4.5)		
Alcohol consumption [n (%)]	30 (14.7)	23 (16.8)	7 (10.4)	2.121	.346
Nondrinkers	168 (82.4)	111 (81.0)	57 (85.1)		
Drinkers	30 (14.7)	23 (16.8)	7 (10.4)		
Former drinkers	6 (2.9)	3 (2.2)	3 (4.5)		
BMI [kg/m^2^, *M* (P25, P75)]	24.79 (22.94, 26.90)	25.04 (22.99, 27.54)	24.19 (22.35, 26.53)	‐1.577	.115
WWI (X̄ ± S)	10.62 ± 1.26	10.77 ± 1.24	10.33 ± 1.26	2.345	.020
ASM [kg, *M* (P25, P75)]	17.11 (15.10, 20.44)	17.62 (15.42, 21.02)	16.30 (14.30, 19.32)	‐2.489	.013
Grip strength [kg, *M* (P25, P75)]	19.90 (16.73, 25.35)	20.90 (17.50, 27.75)	18.80 (16.00, 23.10)	‐2.312	.021

For continuous variables with a normal distribution, an independent samples *t*-test was used; for those with a nonnormal distribution, the Mann–Whitney *U* test (statistic: *Z*) was used; for categorical variables, the *χ*^2^ test (statistic: *χ*^2^) was used.

ASM = skeletal muscle mass of the limbs, BMI = body mass index, *M* (P25, P75) = median (25th percentile, 75th percentile), S = standard deviation, WWI = weight-adjusted-waist index, *X̄* = mean.

### 3.2. Analysis of the correlation between WWI, skeletal muscle mass in the limbs, and grip strength

Stratified Spearman rank correlation tests were utilized (Table 2). In the 60- to 70-year age subgroup, grip strength showed a positive association with ASM (*R* = 0.70, *P* < .001), and WWI showed a positive association with ASM (*r* = 0.44, *P* < .001) and grip strength (*R* = 0.21, *P* = .014). In participants aged >70 years, grip strength remained positively correlated with ASM (*R* = 0.38, *P* = .002); however, WWI showed a negative association with both ASM (*r* = −0.27, *P* = .029) and grip strength (*r* = −0.40, *P* = .001).

**Table 2:** Correlation analysis of skeletal muscle mass, grip strength, and weight-adjusted waist index.

Age group	Variable	Descriptive statistics	ASM	Grip strength	WWI
60–70 Years (n = 137)	ASM	17.62 (15.42–21.02)†	1.000	–	–
	Grip strength	20.90 (17.50–27.75)†	0.700**	1.000	–
	WWI	10.77 ± 1.24‡	0.441**	0.209*	1.000
>70 Years (n = 67)	ASM	16.30 (14.30–19.32)†	1.000	–	–
	Grip strength	18.80 (16.00–23.10)†	0.379**	1.000	–
	WWI	10.33 ± 1.26‡	‐0.267*	‐0.404**	1.000

ASM = skeletal muscle mass, WWI = weight-adjusted-waist index.

† Median (25th percentile, 75th percentile).

‡ Mean ± SD.

* *P* < .05.

** *P* < .01.

### 3.3. Stratified regression analysis of the effect of WWI on grip strength, stratified by age

The results of the stratified regression analysis examining the effect of WWI on grip strength are presented in Table 3. In participants aged 60 to 70 years, model 1 showed a significant positive total effect of WWI on grip strength (*β* = 1.368, 95% CI: 0.375–2.361, *P* = .007), which accounted for 5.2% of the variance. Model 2 analysis revealed a statistically significant positive relationship linking WWI to ASM (*β* = 1.233, 95% CI: 0.747–1.719, *P* < .001), accounting for 15.7% of the ASM variance. In model 3, with both WWI and ASM included simultaneously, the direct effect of WWI became nonsignificant (*β* = −0.371, 95% CI: −1.157 to 0.415, *P* = .352), whereas ASM retained a significant positive predictive effect on grip strength (*β* = 1.410, 95% CI: 1.157–1.663, *P* < .001); model explanatory power increased to *R*^2^ = 0.503.

**Table 3:** Stratified regression analysis of the relationship between weight-adjusted waist index and grip strength.

Age group	Equation	Independent variable	Dependent variable	*R* ^2^	*F*	*β*	SE	*t*	*P*	95% CI
60–70 Years (n = 137)	Mode1	WWI	Grip strength	0.052	7.423	1.368	0.502	2.725	.007	(0.375–2.361)
	Mode2	WWI	ASM	0.157	25.202	1.233	0.246	5.020	<.001	(0.747–1.719)
	Mode3	WWI ASM	Grip strength	0.503	67.875	0.371	0.397	‐0.933	.352	(‐1.157 to 0.415)
						1.410	0.128	11.031	<.001	(1.157–1.663)
>70 Years (n = 67)	Mode1	WWI	Grip strength	0.188	15.061	2.050	0.528	‐3.881	<.001	(‐3.105 to ‐0.995)
	Mode2	WWI	ASM	0.059	4.088	0.727	0.360	‐2.022	.047	(‐1.445 to ‐0.009)
	Mode3	WWI ASM	Grip strength	0.371	18.900	1.542	0.483	‐3.914	.002	(‐2.507 to ‐0.578)
						0.698	0.162	4.318	<.001	(0.375–1.020)

Model 1 tests the total effect c; model 2 tests path a; model 3 tests the direct effect c′ and path b.

ASM = appendicular skeletal muscle mass, CI = confidence interval, WWI = weight-adjusted waist index.

In participants aged >70 years, model 1 showed a significant negative total effect of WWI on grip strength (*β* = −2.050, 95% CI: −3.105 to −0.995, *P* < .001), which accounted for 18.8% of the variance. Model 2 showed a significant negative association between WWI and ASM (*β* = −0.727, 95% CI: −1.445 to −0.009, *P* = .047), accounting for 5.9% of the variance in the ASM. In model 3, the direct effect of WWI remained significantly negative (*β* = −1.542, 95% CI: −2.507 to −0.578, *P* = .002), and ASM showed a significant positive predictive effect on grip strength (*β* = 0.698, 95% CI: 0.375–1.020, *P* < .001); model explanatory power increased to *R*^2^ = 0.371.

### 3.4. Testing for mediating effects

Bootstrapping was employed to assess the mediation effect (Table 4). For the 60- to 70-year age group, ASM demonstrated an indirect effect of 1.739 on the association between WWI and grip strength (95% CI: 1.028–2.611); as the confidence interval excluded 0, ASM exerted a significant mediating effect. The direct WWI effect on grip strength was −0.371 (95% CI: −1.157 to 0.415), which included 0, indicating a nonsignificant direct effect. This pattern is consistent with full mediation (or a suppression effect), whereby WWI influences grip strength indirectly through ASM; the mediated pathway explained 127.1% of the total association.

**Table 4:** Analysis of the mediating effects of body weight-adjusted waist index and grip strength, mediated by appendicular skeletal muscle mass, stratified by age.

Age group	Effect	Effect size (Boot 95% CI)	BootSE	*P*	Relative effect size (%)
60–70 Years group (n = 137)	Total effect	1.368 (0.375–2.361)	0.502	.007	100
	Direct effect	‐0.371 (‐1.157 to 0.415)	0.397	.352	–
	Indirect effect	1.739 (1.028–2.611)	0.400	–	127.1
>70 years (n = 67)	Total effect	‐2.050 (‐3.105 to ‐0.995)	0.528	<.001	100
	Direct effect	‐1.543 (‐2.507 to ‐0.578)	0.483	.002	75.3
	Indirect effect	‐0.507 (‐1.313 to ‐0.006)	0.334	–	24.7

CI = confidence interval.

**Figure 1.**
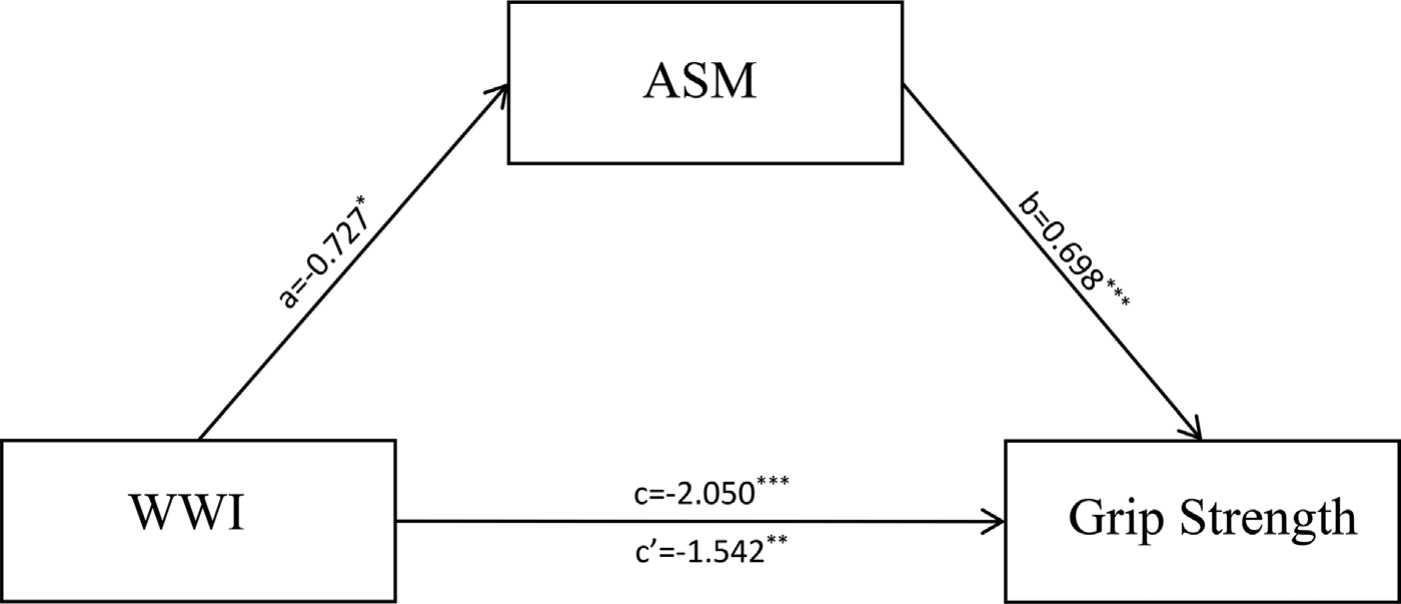
“a” Denotes the effect size of the WWI on appendicular skeletal muscle mass. “b” Denotes the effect size of ASM on grip strength. “c” Denotes the total effect of the WWI on grip strength. “c′” Denotes the direct effect size of the WWI on grip strength. *P < .05, **P < .01, ***P < .001. ASM = appendicular skeletal muscle mass, WWI = weight-adjusted waist index.

In participants aged >70 years, the indirect effect of ASM was −0.507 (95% CI: −1.313 to −0.006); as this interval excluded 0, the mediating effect was significant. The direct effect of WWI on grip strength was −1.542 (95% CI: −2.507 to −0.578), which also excluded 0, indicating a significant direct effect. This pattern is consistent with partial mediation, whereby WWI affects grip strength both directly and indirectly through ASM; the indirect effect accounted for 24.7% of the total effects. Taken together, these results indicate significant age-related heterogeneity in the mediating role of ASM. The mediation pathways are illustrated in Figure 1.

## 4. Discussion

Characterized by concurrent reductions in muscle mass and strength, sarcopenia is a progressive skeletal muscle disorder linked to aging that significantly increases the risk of falls, disability, and mortality among older adults.^[1,11]^ Although diagnostic frameworks for sarcopenia are well established, conventional anthropometric indices have a limited ability to differentiate the distinct contributions of adipose and lean tissues to health outcomes. This limitation underscores the need for more specific indicators to improve early identification and targeted intervention strategies.^[12]^

This investigation represents one of the initial efforts to examine the association between WWI and grip strength among community-dwelling older adults in China, specifically highlighting age-related heterogeneity and mediating mechanisms. Our findings demonstrate an age-dependent shift in the association between WWI and muscle function, with a positive relationship observed in individuals aged 60 to 70 years and a negative association in those aged >70 years. Furthermore, ASM exhibited a variable mediating role, ranging from complete mediation in the younger subgroup to partial mediation in the older subgroup. These results suggest that the predictive value of single anthropometric indices may differ across age groups and highlight the importance of age-stratified evaluation in community-based screenings.

The observed age-related divergence in body composition and muscle function likely reflects the complex biological changes that occur during aging. With advancing age, chronic low-grade inflammation and alterations in neuroendocrine regulation, particularly involving the growth hormone – insulin-like growth factor-1 axis – may promote visceral fat accumulation and accelerate the loss of appendicular muscle mass.^[13,14]^ In addition, lifelong environmental exposure and epigenetic modifications may further impair muscle regenerative capacity.^[15]^ Socioeconomic factors, such as educational attainment, may also indirectly influence lifestyle behaviors and responsiveness to health interventions.^[16]^ However, these mechanisms were not directly assessed in the present study and require further investigation in future studies. The pronounced deterioration in body composition observed among participants aged >70 years likely reflects the cumulative impact of multiple biological and social determinants of health.

Further analysis indicated that the relationship between WWI and muscle health indicators varied substantially by age, suggesting heterogeneous effects of central adiposity at different stages of aging. Among participants aged 60 to 70 years, a higher WWI was positively associated with ASM and grip strength. This finding may be explained by the relatively preserved anabolic hormonal activity and metabolic reserve in this age group, whereby moderate fat accumulation does not exert detrimental effects on muscle function.^[17]^ In contrast, among those aged >70 years, WWI was inversely associated with ASM and grip strength. This transition may reflect a shift toward metabolically adverse fat distribution patterns in advanced age, characterized by increased visceral adiposity, chronic inflammation, and insulin resistance.^[18]^ Nonetheless, these underlying mechanisms were not directly measured and warrant validation in future studies that incorporate relevant biomarkers.

By incorporating WC and adjusting for body weight, WWI offers a more precise assessment of central adiposity in relation to muscle mass than traditional BMI.^[19]^ The age-dependent variation in the WWI–grip strength association observed in this study suggests that the clinical interpretation of WWI should incorporate age-specific considerations. However, appropriate threshold values require confirmation from longitudinal research.

Mediation analysis provides further insight into the pathway linking WWI and grip strength. In individuals aged 60 to 70 years, ASM fully mediated this association, indicating that the effect of WWI on muscle strength primarily operates through muscle mass. These findings align with the well-documented principle that muscle mass serves as a primary determinant of muscular strength.^[20,21]^ In contrast, among participants aged >70 years, ASM only partially mediated the association, with a significant direct effect of WWI on grip strength. This finding suggests that additional factors, such as neuromuscular function, tendon properties, or systemic inflammation, may contribute to the decline in strength in advanced age.^[15,18]^ As these potential mechanisms were beyond the scope of the current investigation, they warrant further exploration in subsequent studies.

From a clinical perspective, these findings support the development of age-specific intervention strategies. For individuals aged 60 to 70 years, interventions targeting the preservation or enhancement of muscle mass, such as resistance training, may be particularly effective in preventing sarcopenia. In contrast, for those aged >70 years, a more comprehensive approach that addresses both muscle mass and functional determinants may be required.^[16]^ Incorporating WWI into routine community assessments may facilitate the early identification of individuals with increased central adiposity who are at risk of functional decline. However, the predictive value of age-specific WWI thresholds remains to be established in prospective studies.^[19]^

Several limitations of this study should be noted. Primarily, the use of a cross-sectional design prevents the determination of causality and restricts the ability to define temporal associations between WWI, ASM, and grip strength. Reverse causality and residual confounding cannot be excluded from this study. Second, the study population consisted predominantly of urban residents (96.1%), and the sample size of participants aged >70 years was relatively small (n = 67), which may limit generalizability. In addition, key covariates, including sex and educational level, were not adjusted for in the mediation analysis, potentially introducing a residual bias. Finally, although BIA is a practical tool for estimating muscle mass, it is less accurate than reference methods such as computed tomography or dual-energy X-ray absorptiometry, which may have resulted in measurement errors.

## 5. Conclusions

To summarize, this research highlights an age-stratified variation in the link between WWI and grip strength within community-dwelling older adults, identifying ASM as a primary mediator. For participants aged 60 to 70 years, ASM substantially explained the association between WWI and grip strength. Conversely, among individuals over 70 years old, WWI showed a direct negative correlation with grip strength, with ASM mediating only a fraction of this impact. These outcomes suggest that WWI could function as a valuable anthropometric tool for detecting risks of muscle function deterioration, especially in advanced aging, where adiposity may influence strength independently of muscle volume. Clinically, strategies aimed at simultaneously reducing fat and preserving muscle are essential to prevent sarcopenia. Ultimately, prospective longitudinal studies encompassing broader and more heterogeneous cohorts are necessary to confirm these results and clarify the causal pathways involved.

## Author contributions

**Data curation:** Yujin Wang, Ying Gao, Yingying Wu.

**Formal analysis:** Yujin Wang, Yingying Wu.

**Methodology:** Yujin Wang, Ying Gao, Yi Li.

**Software:** Yujin Wang, Ying Gao.

**Investigation:** Ying Gao, Yingying Wu.

**Funding acquisition:** Yi Li.

**Supervision:** Yi Li.

**Visualization:** Yi Li.

**Writing – original draft:** Yujin Wang.

**Writing – review & editing:** Ying Gao.
